# Newly diagnosed PRES in a sickle cell diseased patient: a case report

**DOI:** 10.1097/MS9.0000000000000523

**Published:** 2023-04-10

**Authors:** Vaishnavi Gurumurthy, Gauri Jain

**Affiliations:** a Department of PID, Indian Council of Medical Research-NIIH, Mumbai; b Emergency Department, Ashoka Medicover Hospital, Nashik, India

**Keywords:** Acute hypertension, anticonvulsants, PRES, seizure, sickle cell anaemia, sickle cell crisis

## Abstract

Sickle cell disease has many clinical impacts, one such rare finding is systemic hypertension although the literature to support it is debatable. Hypertension along with other key components of sickle cell pathology is one of the reversible causes of posterior reversible encephalopathy syndrome (PRES). Although its triggering factors and pathophysiology is not well documented, hypertension is one of the easily reversible causes of PRES. A well-controlled blood pressure is an aim for reversibility and future recurrence of PRES. However, the addition of other medications like anticonvulsants (levetiracetam and lacosamide) to prevent seizures as a consequence of PRES still remains debatable. Considering the case reported below, the addition of Hydroxyurea to the treatment can be another cause of the recurrence of PRES and needs to be weighed for its risks and benefits.

## Introduction

Highlights

- One unique finding in a sickle cell diseased (SCD) patient was systemic hypertension without pulmonary hypertension.
- Such elevated blood pressure lead to posterior reversible encephalopathy syndrome (PRES) for which the patient got admitted to the hospital.
- After controlling the blood pressure, neurological and radiological improvement was seen which proved the reversibility of PRES.
- Prescribing of hydroxyurea remains debatable as it is one of the causes for PRES, however, beneficial for preventing sickle cell crisis.

A neurological condition known asPRES is typically reversible and presents with a variety of symptoms, including headache, altered mental status, seizures, and loss of consciousness. The term refers to an imaging appearance and symptomatology that may be reversible and is shared by a wide range of conditions, including hypertension, bone marrow transplant, and blood transfusion^1^. The prevalence of hypertension in SCD has been the subject of conflicting reports. A high prevalence of masked hypertension and abnormal circadian patterns have been observed from ambulatory blood pressure monitoring 2,3. Patients with SCD are known to have lower blood pressure due to an unknown aetiology. During sickling and deoxygenation of red blood cells, erythrocyte rigidity and membrane damage cause vascular injury, which is the main cause of hypertension. SCD and high blood pressure raise the risk of stroke and silent cerebral infarction in both children and adults^4^,^5^.

Here, we discuss one such case, in which a young boy with known SCD developed acute hypertension and was diagnosed with PRES and its complications after being checked for a stroke.

## Case history

A 13-year-old boy came in with complaints of body aches and multiple joint pains. He was a known case of sickle cell anaemia, diagnosed at the age of 4 years. There was no history of any blood or bleeding disorders from both maternal and paternal families, although the presence of sickle cell trait is unknown due to non-testing. There were two previous hospitalizations for acute chest syndrome and joint pains. He was on treatment (acetaminophen and folic acid supplement) for the same. For the current episode, he was admitted to a private hospital earlier in view of sickle cell crisis and was transferred to Ashoka Medicover for continuous convulsions which lasted for 20 min. There was no history of blood transfusion done till now for sickle cell disease. On arrival, he was in postictal drowsiness for 20 min and his blood pressure was 150/100 mmHg. He was administered T. Amlodipine 2.5 mg for elevated blood pressure. After coming to the baseline sensorium he was taken for MRI Brain to look for possible causes for convulsions and stroke. He was then admitted to the ICU for partial exchange transfusion for sickle cell crisis when another generalized tonic-clonic convulsion episode occurred. The infusion was stopped and Inj. Midazolam 2 mg IV stat was administered. Seizure activity ceased almost immediately. Post-seizure blood pressure was 180/110 mmHg. Infusion nitroglycerin 25 mg in 50 ml normal saline at 3 ml/h was started in view of continuous high blood pressure. Upon starting nitroglycerin no further seizure activity was noticed. After 24 h infusion of nitroglycerin was stopped and he was started on T. Cilnidipine 5 mg BD, T. Metoprolol 12.5 mg BD, T. Brivaracetam 100 mg BD, T. Clobazam 5 mg HS. On day 2 of post-admission he was diagnosed with PRES by the neurologist. It was concluded that acute hypertension was the trigger for the PRES and he was maintained on antihypertensive medications. His condition improved eventually and was shifted to the general ward by day 4 of post-admission. On discharge after a week of admission, he was prescribed on T. Brivaracetam 100 mg BD, T. Lacosamide 50 mg OD, T. Folic acid 5 mg OD, T. Tramadol+Acetaminophen BD, T. Cilnidipine 5 mg OD. The follow-up after 10 days was uneventful.

## Discussion

Hypertension, immunosuppressive medications like cyclosporine, various antineoplastic agents, severe hypercalcemia, thrombocytopenic syndromes, systemic lupus erythematosus, and various causes of renal failure all share a potentially reversible imaging appearance and symptomatology that is referred to as PRES^1^. The pathophysiology of PRES has been proposed to involve two primary mechanisms:

1. Proteins can eventually pass through the blood–brain barrier as a result of increased arterial pressure. Vasogenic oedema is a typical sign of this disruption.
2. Chemotherapy-affected and septic patients both exhibit endothelial dysfunction. This endothelial damage makes vasogenic oedema possible, which increases vascular permeability and causes additional vasospasm.

When acute neurological symptoms occur in patients with renal failure, fluctuations in blood pressure, use of cytotoxic medications, autoimmune disorders, or eclampsia, a diagnosis of PRES should be considered.

**Figure 1:**
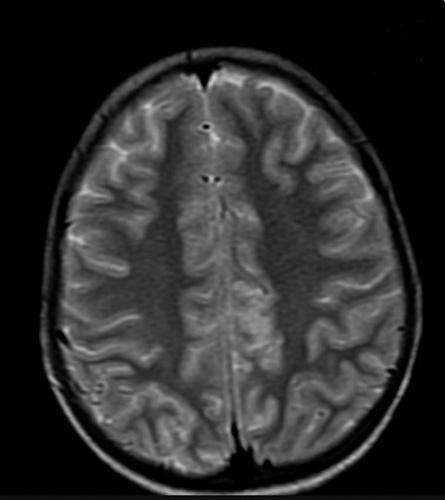
Axial view of MRI Brain showing subtle T2 hyperintensities in left frontal and bilateral parietal cortical sulci and subcortical white matter.

**Figure 2:**
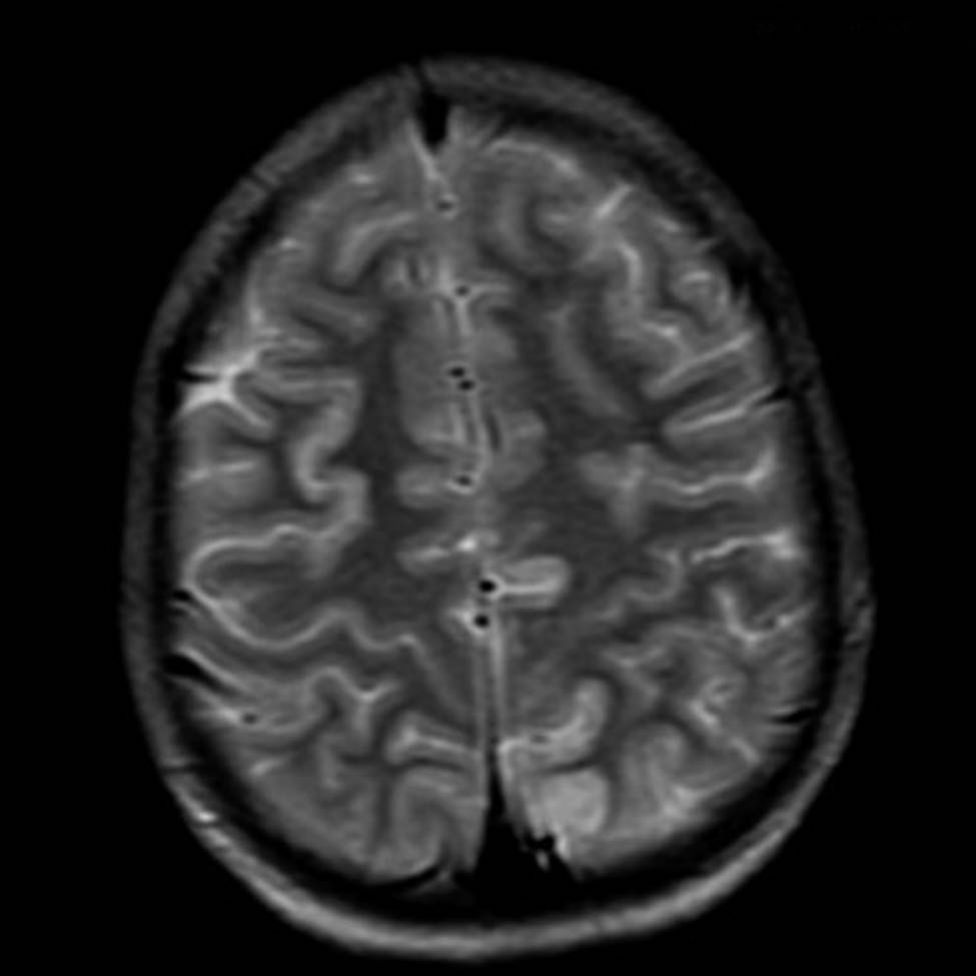
Axial view of MRI Brain showing subtle T2 hyperintensities in left frontal and bilateral parietal cortical sulci and subcortical white matter.

**Figure 3:**
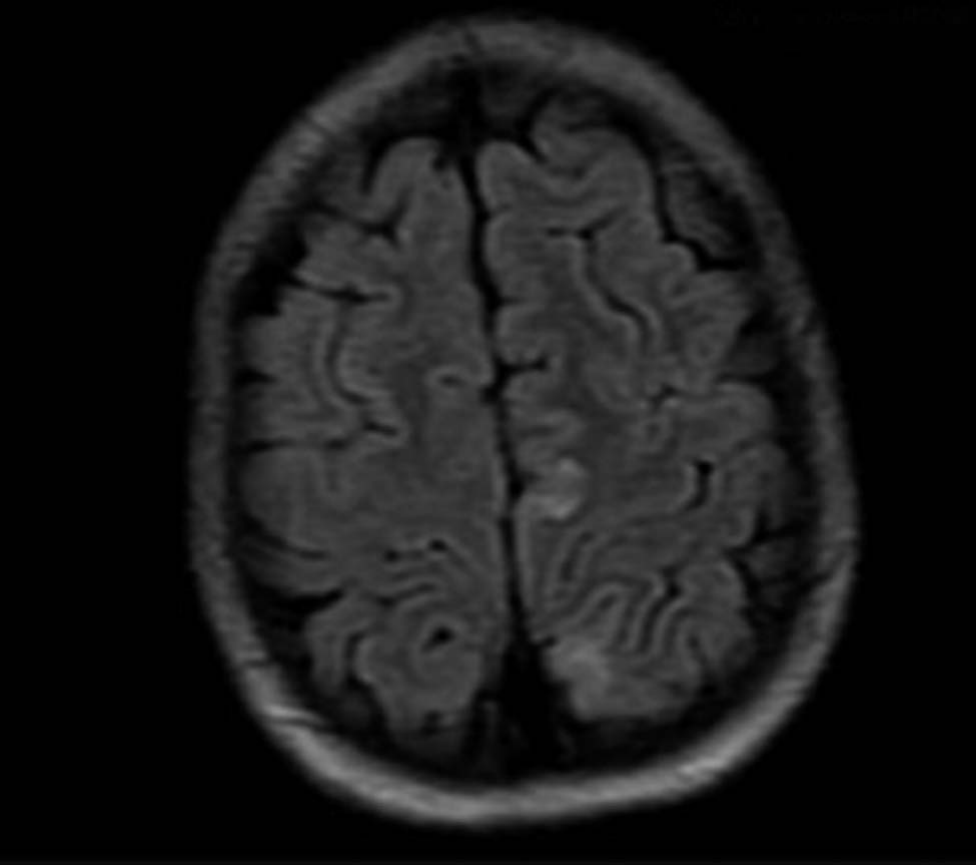
Axial view of MRI Brain showing subtle FLAIR hyperintensities in left frontal and bilateral parietal cortical sulci and subcortical white matter.

It is important to note that the imaging finding in PRES can sometimes mimic those seen in other conditions, such as cerebral oedema, vasculitis, or central nervous system infections so a multidisciplinary approach is necessary for an accurate diagnosis. Bilateral areas of subcortical vasogenic oedema that subside usually within a week are typical radiographic findings. A diagnosis is supported by the presence of haemorrhage, restricted diffusion, contrast enhancement, and vasoconstriction. It’s possible that the syndrome encompasses more symptoms than is typically thought. In its mildest form, this disorder may only present with one clinical symptom—a headache or a seizure—and, in rare instances, radiographs may reveal only a few areas of vasogenic oedema or even normal brain imaging^6^–^8^ (Figures 1–3).

Recent advancements in the treatment of PRES include:

1. Hypertension control: the primary treatment for PRES is to control blood pressure, which is the most common trigger of PRES.
2. Electrolyte management: electrolyte imbalances, such as low levels of calcium, magnesium, and potassium can contribute to PRES and correction of these imbalances is necessary for successful treatment.
3. Vasodilator therapy: vasodilator therapy such as nitroprusside and nimodipine may be used to reduce blood pressure and improve blood flow to the brain in PRES.
4. Plasma exchange: plasma exchange has been used as a treatment for PRES in some cases particularly for those with systemic diseases such as lupus or anti-NMDA receptor encephalitis. Plasma exchange therapy was attempted in this patient, however, in the view of acute sickle cell crisis and not for PRES.
5. Corticosteroids: IV Corticosteroids have been used in some cases of PRES, although their role in the treatment of PRES is still debated.

Most of the time, PRES resolves spontaneously, and patients improve both clinically and radiologically. It is important to note that the treatment of PRES requires a multidisciplinary approach and should be individualised based on the patient’s specific circumstances^9^.

## Conclusion

Although it’s evident the occurrence of hypotension in sickle cell patients, there is a fraction of the patient population who suffer from hypertension and its repercussions. PRES, as previously mentioned, is one such uncommon consequence of acute hypertension. Although the actual pathophysiologic mechanism behind PRES has not yet been identified, it is reversible after the triggering variables have been eliminated. PRES is a distinct entity with distinctive clinical and neuroradiological findings. The major aetiology that is the cause of PRES is often controlled by treatment. Similar to the method used for hypertensive urgency or emergency, the treatment for the aforementioned case aims to lower the raised blood pressures in a controlled setting. In order to reduce the risk of developing ischaemic cerebral illness as a result of extreme blood pressure dropping, a non-rapid decrease in blood pressure is typically aimed after (fall not ˃20%. The target blood pressure being 110/70 mmHg for the above patient). Anticonvulsant drugs (levetiracetam) are occasionally employed as adjuvant therapy, while the best agent, timing, and length of treatment are still debatable. Adding hydroxyurea to the regime still remains unanswered as a way to measure its pros and cons, as it is another cause for precipitating PRES. Indulging into other groups of medications for the prevention of sickle cell crises might be the way to avoid PRES in the future.

## Ethical approval

This work was approved.

## Consent

The consent was taken.

## Source of funding

None declared.

## Conflicts of interest disclosure

None declared.

## Provenance and peer review

Not commissioned, externally peer-reviewed.
